# Explainable Deep Learning Framework for Binary Corrosion Image Classification Using Grad-CAM

**DOI:** 10.3390/s25227070

**Published:** 2025-11-19

**Authors:** Muhammad Amir Imran Aminudin, Mohd Na’im Abdullah, Faizal Mustapha, Kee Kok Eng, Mazli Mustapha, Aliyu Mustapha

**Affiliations:** 1 Department of Aerospace Engineering, Faculty of Engineering, Universiti Putra Malaysia, Serdang 43400, Malaysia; 210237@student.upm.edu.my (M.A.I.A.); faizalms@upm.edu.my (F.M.); 2 Department of Mechanical Engineering, Universiti Teknologi PETRONAS, Seri Iskandar 32610, Malaysia; keekokeng@utp.edu.my; 3 Industrial and Technology Education Department, Federal University of Technology, Minna P.M.B 65, Nigeria; al.mustapha@futminna.edu.ng

**Keywords:** deep learning, convolutional neural networks (CNN), binary classification, corrosion detection, non-destructive testing

## Abstract

Corrosion in metallic materials is a critical challenge in maintenance and safety, and traditional visual inspection methods are often time-consuming, labor-intensive, and dependent on human expertise, highlighting the need for more efficient and reliable solutions. Deep learning, particularly convolutional neural networks (CNNs), provides a promising approach by enabling automated and accurate image-based classification. This study investigates binary image classification of corrosion using four pre-trained CNN architectures, namely ResNet50, MobileNetV2, NASNetMobile, and EfficientNetV2B0, and integrates explainable artificial intelligence (XAI) techniques to provide interpretability and insight into each model’s decision-making process. A curated dataset of 4012 images, divided between corroded and non-corroded surfaces, was pre-processed, and augmented images resulted in a total of 9636 images used to train and evaluate the models. Performance was assessed through accuracy, confusion matrices, computational timing, receiver operating characteristic curves, precision–recall curves, and Cohen’s Kappa. In this paper, Gradient-weighted Class Activation Mapping (Grad-CAM) visualizations are incorporated as an XAI technique to provide interpretable insight into the model’s reasoning process, enabling clear identification of corrosion regions and offering justification for each prediction produced by the system. A key contribution of this work is the integration of Grad-CAM to enhance explainability. The results showed that EfficientNetV2B0 demonstrates stable training with minimal sign overfitting compared to other models. MobileNetV2 achieved the lowest time to train with the datasets given, and ResNet50 achieved the highest classification performance in terms of confusion matrix, with an accuracy of 96.58%. Through Grad-CAM reasoning, EfficientNetV2B0 shows a specific high activation towards corroded regions compared to the other three models that were evaluated.

## 1. Introduction

Corrosion in metallic materials represents a persistent challenge across industries, causing structural degradation, equipment failures, and significant economic losses. Globally, corrosion costs are estimated to consume between 3 and 4% of the gross domestic product (GDP) of industrialized nations, while in aerospace, the U.S. Department of Defense reported annual corrosion-related expenses exceeding 23 billion USD for maintenance and restoration [1]. The problem is equally severe in the oil and gas sector, where localized corrosion in pipelines can compromise safety and reduce service life, leading to costly repairs and environmental risks [2,3]. Traditional inspection approaches, including manual visual inspection and standard non-destructive testing (NDT) techniques such as ultrasonic testing and radiography, remain time-consuming, labor-intensive, and heavily dependent on operator expertise, which limits scalability and consistency [4].

Before the widespread use of artificial intelligence (AI), safety-critical fields relied heavily on algorithmic techniques to enhance detection reliability [5]. These earlier methods laid the groundwork for more advanced approaches. With the emergence of AI and machine learning (ML), new possibilities have arisen for automating corrosion detection. Among the many ML techniques, Artificial Neural Networks (ANNs) are frequently employed because they can capture nonlinear relationships within structured datasets [6]. Nevertheless, traditional ANNs face limitations when applied to image-based problems: they depend on fully connected layers and typically require manual feature engineering. By contrast, Convolutional Neural Networks (CNNs) were developed specifically for vision tasks, enabling automatic extraction of layered spatial features through convolution and pooling. This characteristic makes CNNs particularly effective in corrosion detection, where subtle differences in surface texture and pattern must be recognized with high reliability [7].

Recent research has highlighted the effectiveness of CNN-based methods across different corrosion detection applications. For instance, Reddy and Ramkumar [8] reported that CNNs reached an accuracy of 95.91% in identifying corrosion in pipelines, clearly surpassing the 87.50% achieved by Support Vector Machines (SVMs). In another study, Le [9] explored spiking CNNs in combination with electromagnetic testing probes for corrosion detection in aircraft structures, obtaining accuracies exceeding 93%, with standard CNNs still performing better than SVMs. Likewise, Malashin [3] introduced a CNN-driven system designed to detect pitting corrosion in gas pipelines, demonstrating reliable classification even when lighting conditions varied. Within the aerospace sector, Doğru and colleagues [4] showed that CNNs could support automated inspection of aircraft structures, lowering reliance on manual visual checks while maintaining high detection accuracy.

Beyond conventional applications, researchers have also adapted CNNs to address broader aspects of corrosion analysis. Son et al. [10] integrated CNN models for both corrosion area segmentation and depth estimation, thereby extending the scope of detection beyond simple binary classification. Zhao et al. [11] developed a CNN-based method for corrosion image classification across multiple material types, while still demonstrating strong performance in standard binary classification tasks. In the context of steel structures, Huang et al. [12] applied deep learning to evaluate corrosion severity on Q235 steel and reported that Inception v3 surpassed other CNN architectures with an accuracy exceeding 95%. Similarly, Vu et al. [13] introduced the Lightweight Magnetic Convolutional Neural Network (LMagNet), which required just 90 kB of flash storage and 36 kB of RAM, achieving predictions within 40 ms and proving suitable for mobile deployment. Kong et al. [14] further advanced this field by integrating multi-spectral imaging with CNNs, improving classification reliability under challenging environmental conditions.

Researchers have also examined hybrid and comparative strategies for corrosion-related image analysis. Oyedeji et al. [15], for example, applied CNNs to multiphase corrosion detection and achieved a binary classification accuracy of 94.87% when distinguishing between corroded and non-corroded surfaces. Zhao et al. [11] extended this direction by developing a transfer learning-based CNN model, incorporating fine-tuning on a binary dataset that spanned multiple material types. Their results indicated that the EfficientNetV2 architecture, enhanced through data augmentation and optimized pooling strategies, delivered strong outcomes. EfficientNetV2B0 and EfficientNetV2S reached test accuracies of 0.9176, ROC-AUC values of 0.97, and Precision, Recall, and F1-scores above 0.9. More recently, Farooqui et al. [16] proposed a CNN-based industrial corrosion detection framework that combined traditional CNNs and pretrained models (YOLOv8 and EfficientNetB0), reporting perfect scores (100% accuracy, precision, recall, and F1) on their test dataset.

Beyond classification tasks, several researchers have also turned their attention to interpretability and segmentation methods. Burton et al. [17] investigated the application of Gradient-weighted Class Activation Mapping (Grad-CAM) for corrosion localization. Their study showed that although Grad-CAM could highlight general regions of corrosion, the generated heatmaps were often blurred and failed to accurately outline fine details such as pitting edges or small rust patches. As a result, its usefulness for inspectors was reduced. To overcome this drawback, the authors suggested employing improved variants, including Grad-CAM++ and integrated class activation mapping, which offered higher spatial precision and produced more trustworthy saliency maps. These refinements enhanced both the diagnostic confidence and the overall reliability of CNN-based inspection systems. Rajendran and Subbian [18] noted that although CNNs often achieve high accuracy in corrosion detection, most studies rely on a single architecture or limited case studies with small datasets. They stressed that the lack of systematic comparisons limits generalization and hampers clear guidelines for selecting suitable models. Their review also highlighted the need for standardized datasets, benchmarking, and cross-domain validation to ensure reliable real-world deployment of CNN-based corrosion detection systems.

Despite this progress, significant gaps remain. Many works focus on specific case studies or corrosion types but do not provide a systematic evaluation of multiple CNN architectures under identical conditions for binary corrosion classification. Moreover, issues such as dataset size, variability in environmental conditions, model interpretability, and real-time applicability are often underexplored. Moreover, the application of model explainability techniques, for example, Grad-CAM, remains insufficiently explored within the context of corrosion detection, highlighting a significant gap in the broader development of XAI. In addition to evaluating the classification performance, this study emphasizes the interpretability of the model’s decision-making process through the application of XAI techniques. Specifically, Grad-CAM was employed to visualize the spatial regions that contributed most significantly to the model’s prediction. To address these challenges, the present study investigates binary image classification of corrosion using four pre-trained CNN architectures: ResNet50, MobileNetV2, NASNetMobile, and EfficientNetV2B0. A curated dataset of 4012 images, equally divided between corroded and non-corroded surfaces, was used to train and evaluate the models. Performance was measured using accuracy, confusion matrices, receiver operating characteristic (ROC), and precision–recall (PR) curves, Cohen’s Kappa, and Grad-CAM visualizations for model reasoning, providing a comprehensive comparative evaluation of CNN architectures for corrosion detection.

## 2. Materials and Methods

### 2.1. Research Workflow Overview

**Figure 1:**
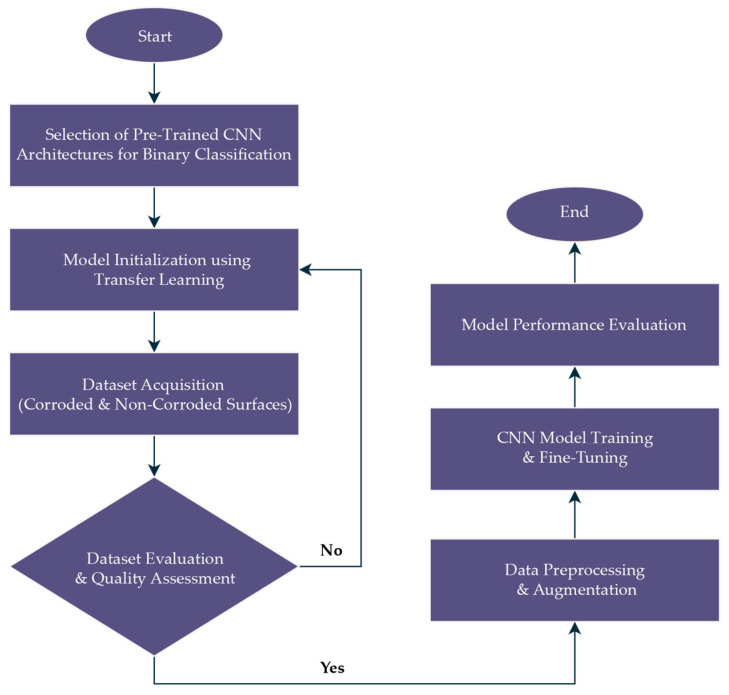
Overall methodological framework for CNN-based binary corrosion classification.

The overall research workflow adopted in this study is summarized in Figure 1. The process begins with the selection of four pre-trained CNN architectures. The next step involved model initialization using transfer learning, where ImageNet-pretrained weights were employed as the starting point to leverage generalized feature representations. This was followed by dataset acquisition, consisting of corroded and non-corroded metallic surface images collected under controlled imaging conditions to ensure dataset reliability. To guarantee data integrity, the dataset underwent evaluation and quality assessment, during which low-quality or ambiguous images were screened and removed. Only images that met predefined quality standards were retained. Once validated, the dataset proceeded to preprocessing and augmentation, which included resizing, normalization, label encoding, and real-time augmentation techniques such as flipping, rotation, and contrast adjustments. These steps were essential to increase dataset diversity and reduce overfitting during model training.

After dataset preparation, the study advanced to CNN model training and fine-tuning. In this stage, transfer learning was refined by unfreezing selected layers of the pre-trained architectures, which allowed domain-specific optimization while still benefiting from the initialization provided by ImageNet.

Finally, a comprehensive model performance evaluation was carried out using multiple quantitative metrics. The combination of quantitative performance metrics and qualitative visualization ensured both statistical robustness and interpretability of the CNN predictions.

### 2.2. Deep Learning Pipeline for Corrosion Classification

**Figure 2:**
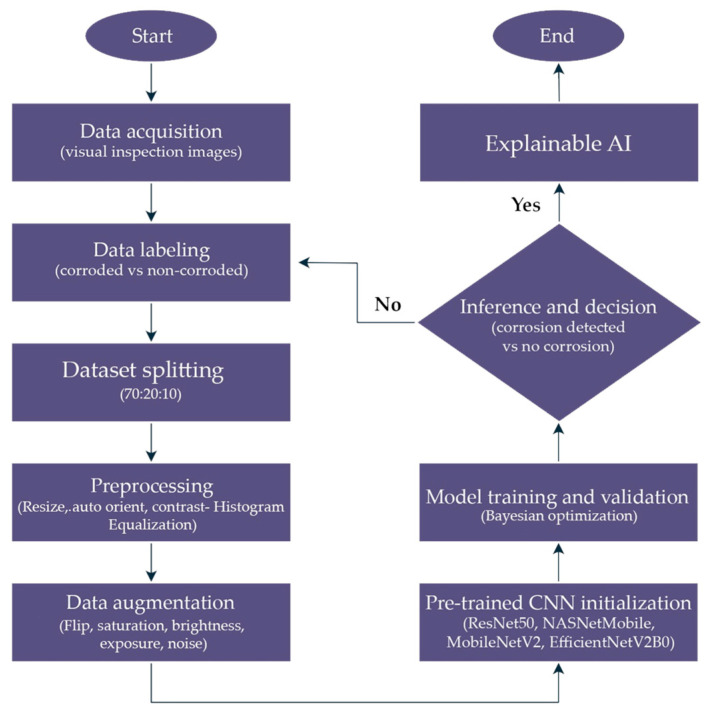
Deep learning pipeline for binary corrosion detection and classification.

To complement the overall research workflow presented earlier, a detailed deep learning pipeline was developed in Figure 2 to illustrate the step-by-step implementation of the binary corrosion classification task. This pipeline outlines the flow from data acquisition and labeling to preprocessing, augmentation, CNN model training with transfer learning, inference for corrosion detection, and final performance evaluation.

The process began by collecting visual images of metallic surfaces, which were manually annotated into two categories (corroded and non-corroded) to enable supervised learning. The dataset was then partitioned into training, validation, and testing groups using a stratified split, ensuring that both classes were proportionally represented across all subsets. This approach reduced sampling bias and provided a more reliable basis for performance evaluation. Preprocessing followed, where all images were resized to 224 × 224 pixels to match CNN input requirements and pixel intensities were normalized to support faster convergence during training. To increase data variability and limit overfitting, augmentation techniques were applied to the training set. These included random rotations, horizontal and vertical flips, adjustments in Hue-Saturation-Value (HSV) color space, and the use of Contrast-Limited Adaptive Histogram Equalization (CLAHE). Such transformations simulated diverse imaging conditions, thereby enhancing model robustness for real-world corrosion detection tasks.

For the model development stage, four pre-trained CNN architectures, namely ResNet50, NASNetMobile, MobileNetV2, and EfficientNetV2B0, were chosen, as each offers distinct advantages in terms of network depth, computational efficiency, and suitability for transfer learning. The models were initialized with ImageNet weights to leverage previously learned visual representations and then fine-tuned for the binary task of corrosion classification. During prediction, each trained network produced probability values indicating the likelihood of corrosion in an input image, which were subsequently compared against a decision threshold to yield a final classification of either “corrosion detected” or “no corrosion.”

In the final stage, the models were evaluated using a combination of statistical measures and interpretability tools. Performance was quantified through several metrics, including accuracy, precision, recall, F1-score, Receiver Operating Characteristic–Area Under the Curve (ROC-AUC), Precision–Recall Area Under the Curve (PR-AUC), and Cohen’s Kappa coefficient. Together, these indicators offered a comprehensive view of classification effectiveness from different perspectives. To complement the numerical results, Gradient-weighted Class Activation Mapping (Grad-CAM) was applied to highlight the image regions most influential in shaping the models’ predictions. This integration of quantitative metrics with visual interpretability provided a rigorous basis for assessing each CNN architecture.

### 2.3. Dataset Description

The dataset comprised visual images of metallic surfaces that were labeled into two categories: corroded and non-corroded. The images have been collected and curated by multiple sources from [19,20,21]. Table 1 shows the curated and split datasets that have been taken for research purposes.

In total, 4012 images were gathered, with 1970 corrosion image samples and 2042 images for no corrosion images. For the 1970 images, the multiple types of corrosion features are summarized in Table 2. All images were retained in the RGB color space, allowing the models to exploit both texture and color variations critical for reliable classification. To prepare the data for training, the set was divided into training, validation, and testing subsets in a 70:20:10 ratio using a stratified split, ensuring that class proportions were consistently preserved across all partitions. The final distribution consisted of 2812 images for training, 821 images for validation, and 398 images for testing. This curated and balanced dataset served as the foundation for subsequent preprocessing, augmentation, and CNN model training.

### 2.4. Hardware and Software Environment

All deep learning experiments were carried out in a controlled computing environment to ensure consistent and stable performance across training sessions. Python served as the primary programming language, with the TensorFlow framework employed for model development and training. A dedicated virtual environment was created to manage dependencies and avoid conflicts between packages. The hardware and software configuration used in this work is outlined in Table 3. The system ran on Windows 11 and was powered by an Intel Core i7-10875H CPU with 16 GB of RAM. For accelerated training, an NVIDIA GeForce RTX 2070 Super Max-Q GPU was employed, utilizing Tensor Cores to enable mixed-precision computation. GPU performance was further supported by the CUDA Toolkit (v7.5) and cuDNN (v10.1), which optimized parallel processing. The environment was managed with Conda (v4.9.2), while Python (v3.8) and TensorFlow (v2.10.0) served as the primary deep learning frameworks.

### 2.5. Data Preprocessing

A series of preprocessing steps were implemented to ensure dataset compatibility with the selected pre-trained CNN architectures and to improve input consistency. The workflow included image resizing, normalization, and binary label encoding. All images were resized to 224 × 224 pixels to conform to the input dimension requirements of ImageNet-pretrained CNNs. This step preserved essential spatial features while minimizing geometric distortions, achieved through center-cropping or padding as necessary. Talebi and Milanfar [22] recommended this resolution, combined with bilinear interpolation, to maintain consistency of spatial features across batches and ensure compatibility with transfer learning models.

Pixel intensity values were normalized to the range (0, 1) by dividing each value by 255. Although most pre-trained architectures apply internal preprocessing, external normalization was essential to accelerate convergence, stabilize numerical computations, and improve the robustness of deep learning models [23]. Images were retained in the RGB color space and converted into NumPy arrays for efficient processing within the TensorFlow/Keras pipeline. Finally, binary label encoding was applied to facilitate supervised learning. Non-corroded samples were assigned a value of 0 and corroded samples a value of 1, corresponding to the sigmoid activation function employed in the output layer of the CNN models.

### 2.6. Data Augmentation

Robust datasets require sufficient variability to capture different aspects of images, thereby enabling models to learn finer details and generalize effectively across diverse conditions. Data augmentation addresses this requirement by artificially increasing dataset diversity through the application of random transformations to input images, which helps mitigate overfitting. As described by Alomar et al. [24], common augmentation techniques include geometric transformations such as flipping, cropping, and random rotations. In addition, color adjustments and saturation have been employed to simulate variations in imaging devices and environmental conditions. Therefore, refer to Table 4 for the detailed specification of the augmentation applied to the datasets.

In this study, augmentation was performed in real time using TensorFlow’s Application Programming Interface (API), specifically the ImageDataGenerator and associated preprocessing pipeline. The validation and testing subsets were not subjected to augmentation to preserve the integrity of the original images and to ensure that model evaluation reflected conditions representative of real-world application scenarios [25]. 

### 2.7. Model Architecture

The architecture selected for this study was chosen because it represents a spectrum of depth, efficiency, and adaptability to transfer learning from ImageNet, allowing for a balanced comparative evaluation in this study [26,27].

ResNet50 is a deep CNN consisting of 50 layers, designed with residual learning through skip connections to overcome the vanishing gradient problem in very deep networks [28]. Its bottleneck structure, which incorporates 1 × 1 convolutions, lowers computational demands while maintaining accuracy, making it suitable for capturing fine-grained texture details [29]. In this work, ResNet50 was used to assess the performance of a deeper architecture in identifying corrosion patterns, with its residual blocks enabling the extraction of subtle structural variations on metallic surfaces.

To balance this with efficiency considerations, lighter architectures such as NASNetMobile were also included, allowing comparisons between high-capacity and resource-efficient models. NASNetMobile is a compact CNN architecture generated through Neural Architecture Search (NAS) with reinforcement learning, which automatically determines an optimized network design [30]. It was developed for mobile and resource-limited applications, offering a balance between computational efficiency and classification accuracy [31]. In this study, NASNetMobile was employed to evaluate the practicality of corrosion detection on platforms with limited resources, where real-time performance is essential.

Alongside this, EfficientNetV2B0 was incorporated to further investigate models that provide both speed and accuracy. EfficientNetV2B0 belongs to the EfficientNetV2 family, designed to strike a balance between training efficiency and predictive accuracy through a combination of fused mobile inverted bottleneck convolution (MBConv) and standard MBConv layers. This hybrid configuration accelerates convergence during training without compromising classification performance [32]. In this study, EfficientNetV2B0 was adopted to benchmark the capability of newer architectures that emphasize both scalability and efficiency in corrosion detection.

By contrast, MobileNetV2 represents a more compact alternative, tailored for highly resource-constrained environments [33]. MobileNetV2 is a lightweight CNN specifically designed for efficient image classification on mobile and embedded platforms [34]. Its architecture incorporates depthwise separable convolutions, linear bottlenecks, and inverted residuals, allowing it to balance predictive accuracy with low computational cost [35]. In this study, MobileNetV2 was employed to examine the capability of highly compact networks, particularly in detecting corrosion with limited sensitivity but minimal resource demands. When considered alongside ResNet50, NASNetMobile, and EfficientNetV2B0, these four models provide a broad comparative framework that spans deep, balanced, and lightweight designs, enabling a comprehensive evaluation of CNNs for binary corrosion detection. To further clarify their individual trade-offs, Table 5 presents a summary of the advantages and limitations associated with each selected architecture.

### 2.8. Mixed Precision and Training Parameter

Mixed precision training is a widely adopted optimization technique for accelerating deep learning workloads, particularly in CNN algorithms, while simultaneously reducing memory consumption [36]. The approach employs two floating-point types with distinct roles: 16-bit (float16) and 32-bit (float32) precision. Float16 is used for computationally intensive operations such as matrix multiplication to enhance processing speed, whereas float32 is retained for numerically sensitive components, including model weights and loss scaling factors, to ensure training stability [37]. Therefore, the mixed precision policy for all pre-trained models has been set to Float 16 or FP16.

All selected pre-trained CNN architectures were fine-tuned using a Bayesian Optimization method, iterated up to 12 times to find a suitable parameter for each of the models to ensure comparison has been well-optimized across models. Training was conducted with a batch size of 32 and the Adam optimizer, which is widely recognized for its stability and efficiency in deep learning. The training was performed for 25 epochs for computational efficiency. Activation functions were model-specific, with ReLU employed in ResNet50, MobileNetV2, and EfficientNetV2B0, while NASNetMobile required a smaller value for stable convergence. The detailed hyperparameter settings for each architecture are summarized in Table 6.

### 2.9. Model Evaluation

The trained models were assessed using multiple quantitative metrics to evaluate the robustness of CNN performance in image classification tasks. Among these, the confusion matrix was employed to provide a structured visualization of classification outcomes, explicitly distinguishing true positives (TP), false positives (FP), true negatives (TN), and false negatives (FN). Accuracy, defined in Equation (1), measures the overall proportion of correctly classified samples. Recall, shown in Equation (2), quantifies the model’s ability to correctly identify positive cases, while Precision, presented in Equation (3), evaluates the proportion of correctly predicted positives relative to all predicted positives. The F1-Score, expressed in Equation (4), represents the harmonic means of Precision and Recall, providing a balanced measure of performance, particularly in cases of class imbalance. This approach enables a more comprehensive understanding of model behavior by highlighting both correct predictions and areas of misclassification, thereby offering deeper insights into classification performance [38]. The formulas of these metrics are as follows:

(1) $$\mathrm{Accuracy}\;=\;\frac{TP+TN}{TP+TN+FP+FN}$$

(2) $$\mathrm{the}\;\mathrm{Recall}=\frac{TP}{TP+FN}$$

(3) $$\mathrm{over}\;\mathrm{Precision}=\frac{TP}{TP+FP}$$

(4) $$F1\;\mathrm{Score}=2\times \frac{Precision\times Recall}{Precision+Recall}$$

In addition to these metrics, model performance was further evaluated using the ROC-AUC and PR-AUC, which provide comprehensive insights into classification performance under varying decision thresholds. Cohen’s Kappa statistics were also employed as a measure of inter-rater agreement between predicted and actual classifications.

(5) $$\mathrm{Cohen}’s\;\mathrm{Kappa},\;𝜅=\frac{e}{e+\frac{I-Ph}{2q0q1}h}$$

Defined in Equation (5), Cohen’s Kappa accounts for agreement occurring by chance, making it a more conservative and reliable evaluation metric than overall accuracy, particularly in cases of imbalanced datasets or noisy labels. The interpretation of Cohen’s Kappa values follows the widely adopted guidelines summarized in Table 7, where values below 0.20 indicate slight agreement, values between 0.41 and 0.60 indicate moderate agreement, and values above 0.80 represent almost perfect agreement [39].

### 2.10. Grad-CAM Visualization Overlay

**Figure 3:**
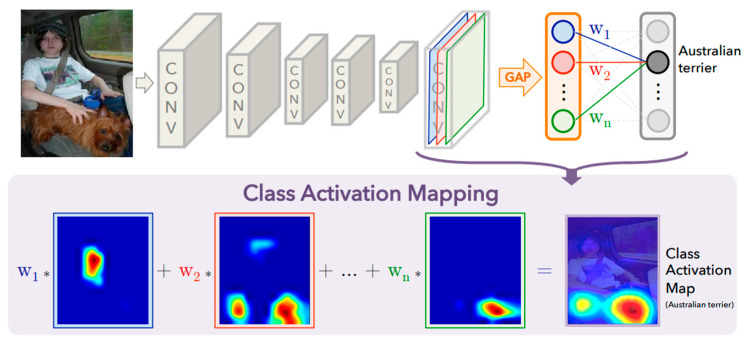
Class activation mapping highlighting the class-specific discriminative regions [41].

To justify the convolutional neural network’s prediction making through image classification, the Gradient-weighted Class Activation (Grad-CAM) was applied in this study for explainable AI for the prediction reasoning. The concept of Grad-CAM, explained by Shen and Huang [40], is based on the gradient of the target class in accordance with the last convolutional layer respective CNN model to determine the most important features in an input image, contributing to the model’s prediction result. Additionally, Grad-CAM can be applied to a vast range of CNN architectures without altering their fundamental structure, particularly effective in transfer learning applications. Then, the gradients are globally average-pooled to compute an importance weight for each feature map in the image. The weighted combination of these maps computed by the models for each feature is then passed through ReLU activation to highlight only positive contributions with respect to the target class. Therefore, the localization map is up-sampled to properly match the size of the input image and generate an overlay as a heatmap, visually indicating the discriminative image regions processed and utilized by the model. Referring to Figure 3, the overview of Class Activation Mapping highlights the mapping by the previous convolutional layer.

Visualization was proven to justify model decision-making by enabling the users to validate whether the CNN is trained specifically to highlight corrosion-specific features, for example, texture irregularities, rust patches, and material discoloration, rather than background noise.

## 3. Results and Discussion

This section presents the model iterations and analysis of each model’s performance. The results focus on the image classification performance and training behavior of the CNN model.

### 3.1. Model Training Plot and Training Time

**Figure 4:**
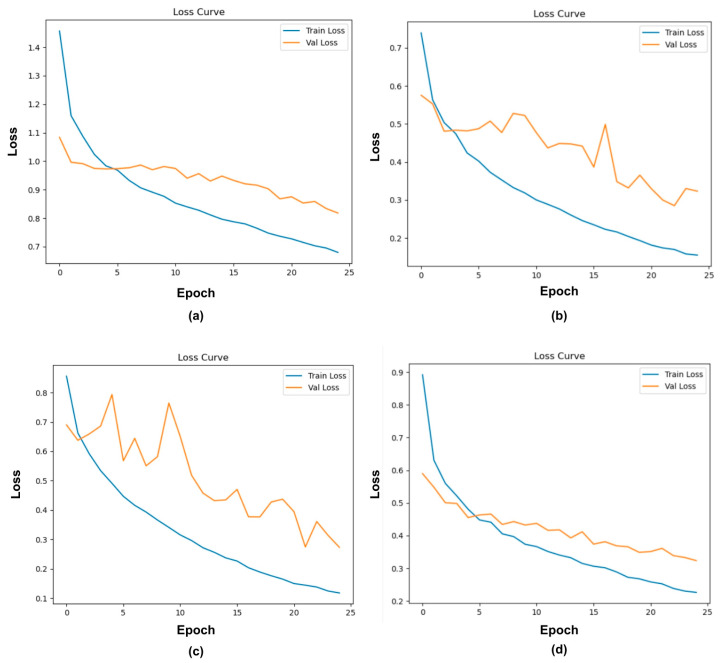
Training loss curves: (**a**) ResNet50; (**b**) MobileNetV2; (**c**) NASNetMobile; (**d**) EfficientNetV2B0.

Figure 4 presents the training and validation loss curves for ResNet50, MobileNetV2, NASNetMobile, and EfficientNetV2B0, providing insights into the learning behavior and generalization performance of each model. In these plots, the blue line denotes the training loss across epochs, while the orange line corresponds to the validation loss, enabling a comparative assessment of model convergence and potential overfitting. Additionally, the horizontal line of the graph displays the epochs, and the vertical line of the graph shows the loss iterated throughout the training phase models.

From Figure 4a, the training loss curve of ResNet50 shows a moderately stable convergence pattern, with steady decline across epochs, and the validation loss shows a slower but consistent downwards trend. Initially, the validation loss plateaus between epochs 5 and 15 before continuing to decline gradually, indicating that despite the model successfully learning from the training data, the generalization process is restrained, reflecting a mild underfitting tendency rather than overfitting. The small but persistent gap between training and validation losses suggests that the model maintains good regularization properties yet has not fully saturated its learning capacity within the observed epoch range. The temporary plateau in validation loss indicates that the model might have benefited from a longer training epoch or adaptive learning-rate decay. This behavior is consistent with previous findings in word recognition tasks, where validation loss stabilized earlier than training loss, highlighting improvements from additional epochs [42].

In Figure 4b, MobileNetV2 illustrates that the training loss exhibits a consistent downward trend across all epochs, converging smoothly toward a minimal value. However, the validation loss follows a similar initial trend, and then plateaus after approximately the tenth epoch, maintaining a higher steady-state level and showing brief fluctuation at an approximate point. This divergence between the training and validation losses is indicative of moderate overfitting, where the network continues to optimize performance on the training set without a corresponding improvement in validation performance. Moreover, this behavior suggests that while the model has successfully captured discriminative features from the training data, at some specific dataset, the model begins to memorize dataset-specific information, beyond the point of generalization. Despite MobileNetV2 being architecturally efficient, the gap between training and validation loss demonstrates a sufficient capacity to overfit when exposed to limited or homogeneous data. The observation can similarly be made when fine-tuning on small or domain-specific datasets, leading to comparable generalization gaps if regularization and augmentation strategies are insufficient [43].

In contrast, Figure 4c demonstrates the training and validation loss curves for NASNetMobile during fine-tuning on the corrosion dataset. The training loss value is 0.1 by epoch 25. In contrast, the validation loss showed considerable reduction throughout the early training stages, oscillating with several local peaks before stabilizing into a slow downward trend after epoch 12. While the validation loss remains higher than the training loss, both curves continue to decrease overall, indicating that the model is still improving and has not yet fully converged. This pattern suggests that NASNetMobile, under the current training setting, is exhibiting mild underfitting rather than overfitting. The model’s gradual and continuous improvement across both training and validation sets indicates that it is an ongoing process of learning the underlying data distribution. However, the gap between the losses implies that the model has not yet achieved complete generalization, and additional training epochs or optimization adjustments may be required to reach convergence. The early volatility of the validation loss points to instability in the learning dynamics, potentially resulting from sensitivity to hyperparameters, for instance, learning rate, batch size, or batch normalization layers. The design relies on Normal Cells, which define feature map size, and Reduction Cells, which downscale feature maps by a factor of two in height and width, thereby enhancing feature representation and efficiency [44].

Figure 4d illustrates a stable and well-aligned relationship between the training and validation loss curves throughout the training process. Both losses decrease consistently with minimal oscillations, indicating a smooth optimization trajectory and strong convergence stability. The validation loss follows the training loss without major divergence, suggesting well generalization performance and minimal overfitting. This behavior highlights EfficientNetV2B0’s architectural efficiency and its capacity to balance model complexity with generalization. The model’s compound scaling approach simultaneously optimizes depth, width, and resolution, enabling it to achieve high detection power without the training instability that is typically experienced in deeper or manually designed architectures. Additionally, the small and consistent generalization gap observed across epochs suggests that the model maintains a favorable bias–variance trade-off, effectively learning a detailed representation rather than memorizing training data.

Table 8 shows the training duration for each CNN model under its best-performing hyperparameter configuration. The results indicate modest variations in computational efficiency among the architectures. MobileNetV2 recorded the shortest training time at 41 min, followed closely by EfficientNetV2B0 at 44 min. ResNet50 required 48 min, while NASNetMobile exhibited the longest training duration at 54 min.

The training time differences reflect the distinct architecture design behind each network. MobileNetV2’s efficiency is due to its lightweight design, utilizing depthwise separable convolutions and inverted residuals to minimize parameter count and computational cost. The lightweight design allows MobileNetV2 to achieve competitive performance with significantly reduced training overhead, ideal for resource-constrained or real-time applications.

The slightly complex EfficientNetV2B0 demonstrates an effective compromise between computational cost and accuracy. The architecture design based on compound scaling approach, jointly optimizes network depth, width, and input resolution, enabling faster convergence without a heavy trade-off to representational power.

In contrast, NASNetMobile’s extended training time reflects the computational overhead introduced by its architecture search-based design. Although optimized through automated Neural Architecture Search, it requires more complex operations and parameter tuning during training. Similarly, ResNet50’s deeper structure and numerous residual blocks contribute to its longer training duration; however, these same elements also provide robust feature extraction and stable gradient propagation.

Overall, the findings highlight that EfficientNetV2B0 offers the most favorable balance between computational efficiency and predictive performance, presenting a similar computational expenditure and being as quick as MobileNetV2 while delivering substantially higher accuracy.

### 3.2. Confusion Matrix

**Figure 5:**
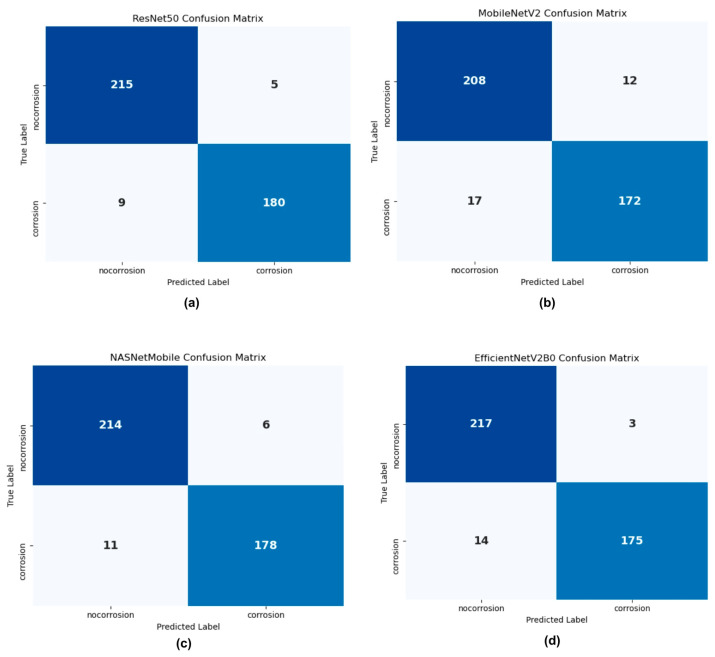
Confusion matrix for (**a**) ResNet50; (**b**) MobileNetV2; (**c**) NASNetMobile; (**d**) EfficientNetV2B0.

The confusion matrices of all four CNN architectures are shown in Figure 5, illustrating their performance on the testing dataset for binary classification of corroded and non-corroded images. These visualizations provide detailed insights into the models’ classification strengths and limitations by quantifying true positives, true negatives, false positives, and false negatives.

Figure 5a shows the classification results of ResNet50, where 215 of 220 non-corroded samples (true negatives) and 180 of 189 corroded samples (true positives) were correctly identified. Misclassifications included two non-corroded images labeled as corroded and nine corroded samples labeled as non-corroded. These results indicate high specificity due to the low number of false positives, although the sensitivity for detecting corrosion textures was slightly reduced [45].

Figure 5b illustrates the performance of MobileNetV2, which correctly classified 208 non-corroded samples and then achieved 172 correct classifications for corroded samples. This reduced sensitivity reflects the trade-off inherent in its lightweight design, where computational efficiency is prioritized at the expense of capturing subtle corrosion features under challenging conditions.

In contrast, Figure 5c presents the confusion matrix for NASNetMobile, which correctly classifies 214 non-corroded and 178 corroded samples. Its cell-based architecture, derived from Neural Architecture Search, demonstrated strong adaptability to image texture variations, enabling superior performance in distinguishing between corroded and non-corroded cases.

Finally, Figure 5d highlights the performance of EfficientNetV2B0, with 217 non-corroded and 175 corroded samples correctly classified. The integration of fused-MBConv layers and progressive resizing strategies contributed to the model’s generalization ability, resulting in robust overall performance across both categories. Table 9 presents the overall evaluation results obtained from Equations (1)–(4), which define accuracy, precision, recall, and F1-score.

ResNet50 achieved the best overall performance among the four architectures, with the highest accuracy (0.9658) and F1-score (0.9626), supported by strong recall (0.9524) and precision (0.9730). These results demonstrate its ability to maintain a balanced trade-off between sensitivity and specificity, attributed to its Residual Network design that adapts effectively to dataset variations. Similar behavior has been reported in automated COVID-19 detection using Computed Tomography (CT) scans, where ResNet50 achieved accuracy, recall, precision, and F1-score values of 0.8852, 0.8840, 0.8800, and 0.8740, respectively [46].

EfficientNetV2B0 ranked second, attaining an accuracy of 0.9584 and the highest precision among the models (0.9831), indicating its reliability in minimizing false positive predictions. Its recall value of 0.9259 further reflects robust sensitivity to corrosion features. These results are consistent with prior research on bird classification tasks, where EfficientNetV2B0 achieved accuracy, precision, recall, and F1-score values of 0.912, 0.905, 0.910, and 0.907, respectively [47].

NASNetMobile followed similar with an accuracy of 0.9584 and precision of 0.9674, while its recall (0.9418) was lower compared to ResNet50 and higher than EfficientNetV2B0. This indicates that Neural Architecture design excelled in reducing false positives but exhibited a tendency to miss some corroded cases, reflecting a slight bias toward non-corroded classifications. Previous work on weather image classification reported that NASNetMobile achieved accuracies of up to 0.90 when handling four-class classification tasks [48], which reinforces its suitability for binary corrosion detection in this study.

MobileNetV2 recorded the lowest performance, with an accuracy of 0.9291 and an F1-score of 0.9223. Although it maintained relatively good precision (0.9348), its recall (0.9101) was slightly lower than the other models, indicating reduced sensitivity to corrosion detection. This underperformance can be attributed to its lightweight design, which emphasizes computational efficiency at the expense of representational capacity. Nonetheless, the results succeed those observed in prior studies, where MobileNetV2 achieved accuracy, precision, recall, and F1-score values of 0.80, 0.83, 0.79, and 0.85 in more complex multi-class classification tasks [49].

Comparatively, ResNet50 achieved the best balance of sensitivity and specificity, correctly detecting most corroded and non-corroded samples, while ResNet50 demonstrated high specificity but slightly weaker sensitivity. EfficientNetV2B0 offered stable generalization with moderate trade-offs, whereas MobileNetV2 showed the lowest sensitivity, reflecting its lightweight design constraints. These findings align with prior literature where lightweight models such as MobileNet often compromise sensitivity for efficiency [50], while deeper or NAS-based architectures demonstrate superior adaptability in defect detection tasks [51]. Similar results were also reported by Das et al. [52] who achieved higher sensitivity with EfficientNet variants for corrosion detection, reinforcing the suitability of these architectures for non-destructive testing applications.

### 3.3. Receiver Operating Characteristics (ROC)

**Figure 6:**
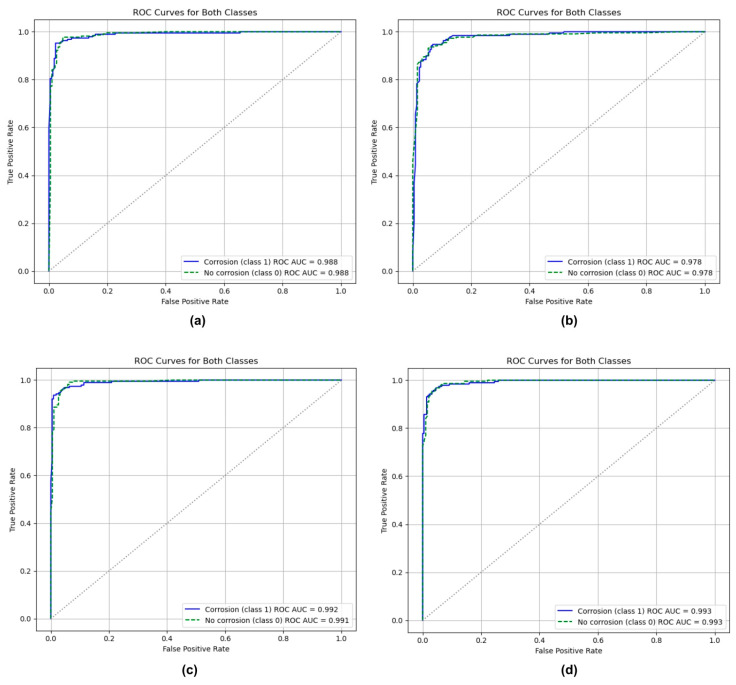
Receiver operating characteristics for (**a**) ResNet50; (**b**) MobileNetV2; (**c**) NASNetMobile; (**d**) EfficientNetV2B0.

Figure 6 illustrates the ROC curves and corresponding AUC values for the four CNN architectures, providing a comparison of their ability to distinguish between corroded and non-corroded surfaces. 

As shown in Figure 6a, ResNet50 achieved an AUC of 0.988 for both classes, reflecting its strong capacity to maintain a high true positive rate (TPR) while minimizing the false positive rate (FPR). Such performance is consistent with findings from previous studies on automated image classification, where multi-class models reported lower AUC values of approximately 0.9342 [53]. The ROC curve for both classes exhibits a steep rise toward the top-left corner, suggesting a high true positive rate with minimal false positives. This observation can be understood as a well-generalized model that has learned robust feature representations rather than memorizing training data. The curves showed a steep rise for both classes toward the top-left corner, signifying a high true positive rate with minimal false positives. Near-perfect AUC performance achieved by ResNet50 can be attributed to its deep residual learning architecture, which facilitates efficient gradient propagation and enables the extraction of highly discriminative features.

MobileNetV2, presented in Figure 6b, attained an AUC of 0.978. Although slightly lower than ResNet50, this value demonstrates a reasonable trade-off between efficiency and performance. Quantitatively, the computed AUC values for both classes are robust, with a score of 0.978, which implies that in 97.8% of all possible random pairings of corrosion and non-corrosion samples, the model correctly assigns a higher probability to the true corrosion instance. The near-identical AUC scores between both classes suggest that the model does not exhibit class bias, reflecting balanced learning and consistent feature extraction across the dataset. Its architecture, characterized by inverted residual block and depthwise separable convolutions, appears to effectively capture fine-grained texture irregularities and oxidation patterns on metallic surfaces. Comparable research has reported that MobileNetV2 achieved an AUC of 0.9260 in other classification tasks [54], underscoring its effectiveness despite being lightweight.

Figure 6c shows NASNetMobile, which achieved an AUC of 0.992 for corrosion and 0.991 for non-corrosion cases. These results indicate near-perfect classification capability, confirming that the NASNetMobile model possesses excellent discriminative power between corroded and non-corroded regions. The scores above 0.99 suggest an almost ideal separation between positive and negative instances. Therefore, the NASNetMobile model demonstrates an extremely high reliability in predicting corrosion presence with minimal overlap between the two probability distributions. NASNetMobile’s performance advantage can be attributed to its Neural Architecture Search (NAS) optimized design, which adaptively assembles convolutional cells to achieve an efficient feature extraction with minimal redundancy.

Finally, EfficientNetV2B0 achieved an AUC of 0.993 for both classes, reflecting an exceptionally strong discriminative ability, as shown in Figure 6d. In practical terms, the model correctly identifies and differentiates corroded surfaces from non-corroded ones with near-total reliability. An AUC score above 0.99 is generally considered outstanding, implying that the EfficientNetV2B0 classifier is utilized with near-perfect separation between the two categories. This high AUC demonstrates the model’s strong generalization, suggesting it performs effectively not only on the training data but also on previously unseen samples. EfficientNetV2B0’s excellent performance can be factored to its compound scaling approach, which systematically balances the model’s depth, width, and input resolution. The inclusion of squeeze-and-excitation and fused-MBConv layers enhances its ability to focus on informative features and capture subtle texture variations associated with corrosion, for example, pitting, roughness, and color changes. These results are demonstrated with recent studies where EfficientNetV2B0, with partial fine-tuning (20% unfreezing), achieved an AUC of 0.97 [55], confirming its effectiveness for high-precision classification tasks.

In summary, all four CNN architectures demonstrated strong discriminative ability with AUC values exceeding 0.96, demonstrating their reliability in differentiating corroded from non-corroded surfaces. Among them, EfficientNetV2B0 and ResNet50 both scored 0.993 and 0.988, respectively, and consistently achieved the highest classification performance, reflecting their superior feature extraction depth and robustness in handling complex corrosion patterns. NASNetMobile also produced highly competitive results, emphasizing the effectiveness of Neural Architecture Search (NAS) in optimizing model topology for corrosion detection tasks. Although MobileNetV2 scored slightly lower than the rest, it remains a strong candidate for real-time or edge-based deployment, offering an effective balance between accuracy and computational efficiency.

### 3.4. Precision–Recall Curve

**Figure 7:**
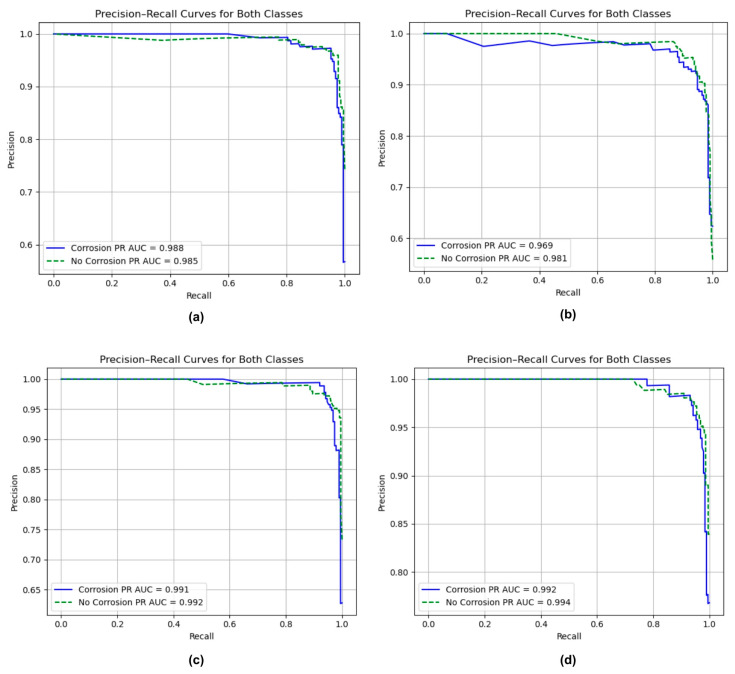
Precision–recall curve for (**a**) ResNet50; (**b**) MobileNetV2; (**c**) NASNetMobile; (**d**) EfficientNetV2B0.

Figure 7 illustrates the ROC curves and corresponding AUC values for the four CNN architectures, providing a comparison of their ability to distinguish between corroded and non-corroded surfaces. PR curves are particularly useful when evaluating classification tasks with class imbalance, as they provide insights into the trade-off between precision and recall across different thresholds.

In Figure 7a, both ResNet50 curves for the corrosion and non-corrosion classes remain consistently high across the recall range. The model achieves PR AUC scores of 0.988 for corrosion and 0.985 for non-corrosion, which indicates excellent overall precision–recall balance. These results suggest that ResNet50 correctly identifies nearly all corrosion cases while maintaining minimum potential false alarms. The consistently high precision across most recall levels reflects a model that not only detects corrosion accurately but also produces predictions that can be trusted in practical inspection settings. The curves remain close to the upper boundary of the plot, indicating that ResNet50 maintains high prediction confidence even as the detection threshold is adjusted. A slight decline in precision near the highest recall values is expected, as capturing the last few true positives often introduces a small number of false positives. However, this trade-off is minimal and does not meaningfully impact the overall classification performance. Figure 7b demonstrates that the model achieved a PR AUC of 0.969 for the corrosion class and 0.981 for the non-corrosion class. These values reflect a high level of discriminative capability, confirming that MobileNetV2 can effectively differentiate between corroded and non-corroded surfaces. Although its PR AUC values are slightly lower than those of deeper architectures, for instance, ResNet50 or EfficientNetV2B0, they remain within the range typically associated with strong classification performance. A PR AUC exceeding 0.95 indicates that the model is both precise and sensitive, maintaining reliable detection with a low incidence of misclassification. Based on the curve shape, the PR curves demonstrate consistently high precision across most recall values, remaining close to 1.0 until recall exceeds approximately 0.9. However, the slight decline in precision at higher recall levels suggests a moderate increase in false positives as the model prioritizes the detection of all possible corrosion regions. This trade-off is common in practical inspection tasks, where capturing every potential instance of corrosion is more critical than completely avoiding false alarms. Therefore, MobileNetV2 exhibits strong practical utility in automated corrosion detection systems where accurate and efficient operation is required.

Figure 7c illustrates NASNetMobile’s precision–recall curves for both corrosion and non-corrosion classes. The model achieves high PR-AUC values of 0.991 and 0.992 for corrosion and non-corrosion, respectively, reflecting outstanding precision and recall balance across both categories. These results indicate that NASNetMobile is highly capable of maintaining accurate classifications, even as the decision threshold varies, effectively minimizing false positives and false negatives throughout the evaluation range. The curves for both classes remain consistently close to the upper-right corner, indicating that the model sustains high precision across almost the entire recall spectrum. This performance is particularly significant for corrosion detection tasks, where missing corroded regions or misclassifying intact surfaces can lead to costly maintenance decisions. The near-identical AUC values also suggest balanced generalization between the two classes, showing that the model does not favor one class over the other. Finally, Figure 7d shows the EfficientNetV2B0 PR curves for both corrosion and non-corrosion classes. The model demonstrates good PR-AUC scores of 0.992 for corrosion and 0.994 for no-corrosion, reflecting a robust ability to sustain high precision while achieving strong recall across a range of classification thresholds. This outcome indicates that the model maintains a well-balanced trade-off between correctly identifying corroded regions and minimizing false alarms. The close alignment of the two curves, both remaining within the upper-right boundary of the graph, underscores consistent and unbiased model behavior. The symmetrically aligned curve suggests that EfficientNetV2B0 effectively generalizes across both classes without a tendency to favor one class over another.

Overall, all four models demonstrated strong classification performance with PR-AUC values exceeding 0.96, demonstrating strong discriminative performance across both classes. Among them, EfficientNetV2B0 attained the highest PR-AUC values of 0.992 and 0.994 for corroded and non-corroded cases, respectively, closely followed by NASNetMobile’s 0.991 and 0.992, ResNet50’s 0.998 and 0.985, and MobileNetV2’s 0.969 and 0.981. These results align with the previously observed ROC-AUC trends, indicating the robust generalization and reliability of EfficientNetV2B0 as the most effective architecture for corrosion detection within this study.

### 3.5. Cohen’s Kappa Evaluation

Table 10 presents the Cohen’s Kappa values obtained for each model, providing an assessment of inter-rater reliability or agreement between predicted and actual classifications. The interpretation of these values follows the criteria outlined in Table 6, where each range corresponds to a specific level of inter-rater agreement. Notably, NASNetMobile and EfficientNetV2B0 achieved values above 0.9162 and 0.9160, respectively, indicating strong to almost perfect agreement, whereas MobileNetV2 produced a comparatively lower value, reflecting only moderate to strong agreement. From Table 10, ResNet50 achieved the highest Cohen’s Kappa value of 0.9310, which falls within the category of almost perfect agreement, as defined in Table 7. This result indicates that NASNetMobile consistently provides reliable classification between corroded and non-corroded cases. Its superior performance aligns with the high precision–recall and ROC–AUC metrics previously reported in this study, further validating the robustness of NAS-based architectures. Similar findings were reported by Walid et al. [56], where NASNetMobile achieved a Kappa value of 0.8674 in a three-class classification problem, highlighting its adaptability and strong generalization capability across diverse tasks. The ability of Neural Architecture Research (NAS) to automatically optimize architectural design likely contributes to its superior inter-rater reliability in corrosion detection.

EfficientNetV2B0 followed closely, attaining a Kappa value of 0.9160, which also corresponds to almost perfect agreement. This demonstrates the architecture’s consistent predictive reliability across different corrosion cases. Walid et al. [56] reported a comparable Kappa value of 0.85 when applying EfficientNetV2B0 in a similar classification task, supporting the present study’s findings. The use of fused-MBConv layers and progressive learning strategies appears to enable EfficientNetV2B0 to maintain both stability and efficiency, making it a strong alternative to NASNetMobile, particularly when computational resources are limited.

ResNet50 achieved a Kappa value of 0.9310, which also falls into the almost perfect agreement range. Its residual learning framework, characterized by skip connections, contributes to stable gradient propagation and effective training in deep architectures. However, the slightly lower agreement compared to NASNetMobile and EfficientNetV2B0 suggests occasional misclassifications, especially in borderline corrosion cases where texture patterns are subtle. Previous research on ResNet50 in medical imaging classification similarly reported strong but slightly lower inter-rater reliability compared to newer architectures [57], supporting this observation.

In contrast, MobileNetV2 recorded the lowest Kappa value of 0.8571, which is interpreted as substantial agreement. While its lightweight design allows for fast and resource-efficient inference, the trade-off comes at the expense of slightly reduced reliability compared to the other three models. This is consistent with prior studies, such as Howard et al. [58]) and Sandler et al. [59], which highlighted that MobileNetV2’s efficiency-oriented architecture tends to sacrifice sensitivity in complex classification tasks. Overall, the results indicate that ResNet50 and EfficientNetV2B0 are the most reliable architectures for research purposes due to their strong accuracy, Kappa values, and balanced performance, while MobileNetV2, despite its lower sensitivity, remains well suited for real-world applications where computational efficiency and rapid inference are prioritized.

### 3.6. GradCam Interpretation

**Figure 8:**
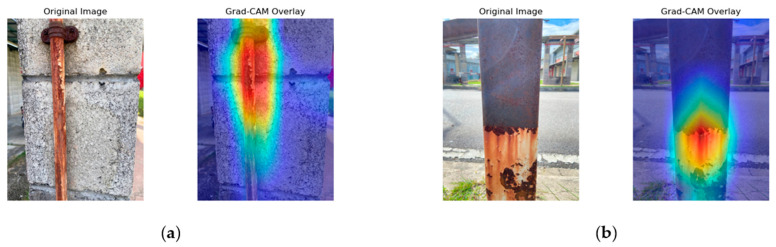
ResNet50 Grad-CAM illustration. (**a**) Activation focused on corrosion around the bolt head and vertical streak. (**b**) Activation highlighting severe corrosion at the lower section of the metal column.

**Figure 9:**
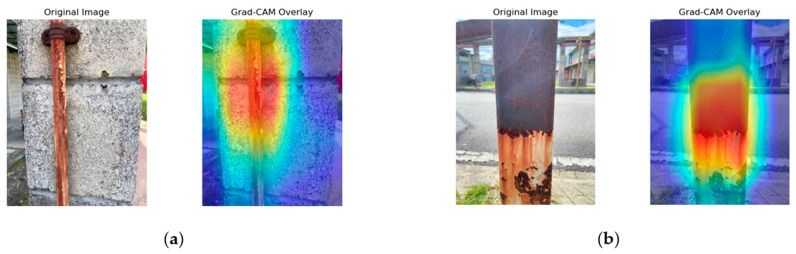
MobileNetV2 Grad-CAM illustration. (**a**) Activation focused on corrosion around the bolt head and vertical streak. (**b**) Activation highlighting severe corrosion at the lower section of the metal column.

**Figure 10:**
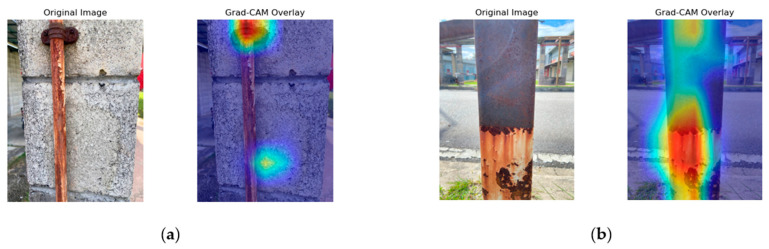
NASNetMobile Grad-CAM illustration. (**a**) Activation focused on corrosion around the bolt head and vertical streak. (**b**) Activation highlighting severe corrosion at the lower section of the metal column.

**Figure 11:**
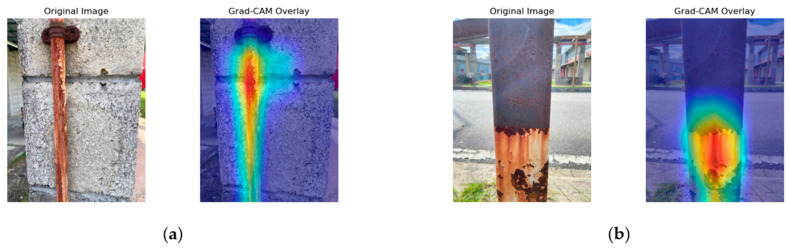
EfficientNetV2B0 Grad-CAM illustration. (**a**) Activation focused on corrosion around the bolt head and vertical streak. (**b**) Activation highlighting severe corrosion at the lower section of the metal column.

Figure 8, Figure 9, Figure 10 and Figure 11 present the Grad-CAM overlays generated by ResNet50, MobileNetV2, NASNetMobile, and EfficientNetV2B0 for corrosion detection. These visualizations highlight the specific regions of the images that contributed most to the models’ predictions, thereby offering transparent and interpretable insights into the decision-making process of the deep learning architectures.

Referring to Figure 8, the Grad-CAM overlays generated by ResNet50 highlight the corroded metal surfaces. In Figure 8a, the activation is concentrated around the corroded bolt head and the vertical corroded region extending downward along the metal surface. ResNet50 demonstrates strong localization capability by focusing on galvanic corrosion, discoloration, and surface degradation patterns while maintaining minimal activation in the background. The attention maps further reveal that the model effectively captures textural irregularities, particularly at the bolt connection point where oxidation is most severe. In Figure 8b, the overlays indicate extensive corrosion detection on the metal column, with high activation intensity localized at the heavily corroded lower section, potentially corresponding to pitting or penetration corrosion. Although the model captures the irregular corrosion boundaries with high fidelity, it underrepresents the upper region where uniform corrosion is present. Nevertheless, the discriminative ability is evident, as the background remains largely inactive.

Figure 9 illustrates the Grad-CAM results of MobileNetV2. In Figure 9a, the activation is strongly localized around the corroded bolt and surrounding corrosion patterns, with minimal spillovers into the background. This reflects the model’s capacity to discriminate between corroded from non-corroded regions effectively, although slight activation in the concrete background is observed. Figure 9b shows broader and more diffuse activation patterns concentrated on the lower corroded section of the metal bar, extending vertically to encompass the corrosion boundary. While MobileNetV2 effectively identifies the extent of surface degradation, the diffuse nature of the heatmap and occasional spillover into adjacent areas suggest higher sensitivity and reduced precision compared to deeper models, consistent with its lightweight architecture.

NASNetMobile’s Grad-CAM visualizations, as shown in Figure 10, demonstrate focused and accurate localization. In Figure 10a, the activation is concentrated around the corroded bolt head and the vertical corroded surface. This suggests that the model effectively targets the most critical regions, particularly where galvanic corrosion is most pronounced. However, minor scattered activation appears in the lower concrete background, indicating some sensitivity to contextual features beyond the immediate corrosion zone. In Figure 10b, the overlays display intense and well-defined activation around the lower corroded region, capturing irregular corrosion boundaries with precision. The ability to localize corroded areas while minimizing background activation highlights NASNetMobile’s strong interpretability and detection performance.

EfficientNetV2B0 Grad-CAM overlays are shown in Figure 11. In Figure 11a, the attention patterns are diffused but extend consistently along the corroded metal surface, capturing corrosion details and the geometry of the subject. However, activation extends noticeably into background regions, suggesting reduced localization sharpness. Figure 11b presents overlays of extensive corrosion damage on the metal column, where activation is centered on the lower corroded section with consistent and predictable patterns. While EfficientNetV2B0 effectively highlights corroded boundaries and surface irregularities, the spread of activation into the background indicates a trade-off between generalization and precise localization.

In summary, the Grad-CAM visualizations reveal distinct localization behaviors across the four models. NASNetMobile demonstrated the most precise and well-defined activation patterns, effectively isolating corroded regions with minimal background interference. ResNet50 also achieved good localization but occasionally underrepresented uniform corrosion areas. EfficientNetV2B0 captured broader corrosion boundaries and structural details, although with some spillovers into background regions. MobileNetV2 showed reasonable discrimination but exhibited more diffuse attention, reflecting the trade-off between its lightweight design and localization accuracy.

## 4. Conclusions

This study confirms the feasibility of employing CNNs for automated corrosion detection in metallic surfaces through binary image classification. A balanced dataset of 710 images was analyzed using four pre-trained architectures (ResNet50, MobileNetV2, NASNetMobile, and EfficientNetV2B0), which were evaluated through a comprehensive suite of metrics, including accuracy, precision, recall, F1-score, ROC-AUC, PR-AUC, Cohen’s Kappa, and Grad-CAM visualizations for justifying the prediction made by the model.

The results highlight both the strengths and trade-offs of the evaluated models. NASNetMobile emerged as the best-performing architecture, achieving the highest classification accuracy and offering strong interpretability through precise attention maps, thereby demonstrating its suitability for research-oriented applications. EfficientNetV2B0 distinguished itself by delivering superior precision and robust generalization, reflecting the advantages of its hybrid Fused-MBConv design in handling diverse corrosion patterns. ResNet50 provided a well-balanced performance profile, leveraging residual learning to maintain stability in deeper networks, although its relatively lower recall suggests challenges in detecting certain corrosion cases. MobileNetV2, while less sensitive overall, demonstrated notable efficiency and rapid inference, underscoring its novelty as a lightweight architecture particularly suited for deployment in resource-constrained environments such as portable inspection devices or UAV-based monitoring systems.

Beyond quantitative results, the Grad-CAM overlays offered critical interpretability by visualizing model attention on corroded regions. NASNetMobile exhibited precise localization of corrosion features, while EfficientNetV2B0 effectively captured broader structural boundaries. These findings underscore the potential of CNN-based methods to strengthen NDT practices by reducing human error, improving consistency, and lowering inspection costs when compared to traditional visual inspection methods that remain labor-intensive and operator-dependent.

Future research should focus on advancing these methods beyond binary classification toward object detection and real-time monitoring, enabling automated localization from live camera feeds. Expanding the scope of detection to include specific corrosion types, together with the development of larger and more diverse datasets, will be essential to enhance model robustness and generalizability under real-world conditions. Collectively, these directions chart a path toward practical deployment of CNN-driven corrosion detection systems that can transform industrial inspection workflows and improve structural safety.

## Figures and Tables

**Table 1:** Dataset source composition.

Datasets Source	Corrosion	No Corrosion	Total
[19]	919	958	1877
[20]	751	784	1535
[21]	300	300	600

**Table 2:** Types of corrosion features images.

Source	Pitting	Crevice	Stress	Uniform
[19]	300	100	59	460
[20]	250	90	51	360
[21]	80	46	48	126

**Table 3:** Hardware and software specifications for experimental setup.

Component	Specification
CPU	Intel Core i7-10875H
GPU	NVIDIA RTX 2070 Super Max-Q
RAM	16 GB
Operating System	Windows 11
Python	3.8
Tensorflow	2.10.0
CUDA Toolkit	7.5
cuDNN	10.1
Virtual manager	Conda 4.9.2

**Table 4:** Augmentation type and parameters.

Augmentation Type	Parameters
Flip	Horizontal, vertical
Saturation	−20% to +20%
Brightness	−15% to +15%
Exposure	−5% to +5%
Noise	0.22% pixels

**Table 5:** Advantages and disadvantages of selected CNN architectures in this study.

Model	Advantages	Disadvantages	Reference
ResNet50	Deep 50-layer architecture with residual learning helps mitigate vanishing gradient problems. Strong extraction capability, effective for capturing fine-grained corrosion textures.	Computationally intensive, requiring higher training time and GPU memory. May overfit small datasets without augmentation.	[26]
NASNetMobile	High computational efficiency, suitable for mobile or embedded applications. Good balance between speed and accuracy in resource-constrained environments.	Accuracy generally lower than deeper networks. May struggle with highly complex corrosion features compared to larger models.	[30,31]
EfficientNetV2B0	Strong trade-off between accuracy, scalability, and efficiency. Better convergence with fewer parameters compared to older CNNs.	Architecture more complex to implement and tune. Requires careful fine-tuning for small datasets.	[32,33]
MobileNetV2	Employs depthwise separable convolutions and inverted residuals for reduced complexity. Suitable for deployment in real-time inspection systems.	Lower sensitivity and recall compared to deeper models. May miss subtle corrosion features in noisy environments.	[34,35,36]

**Table 6:** Training hyperparameters after Bayesian optimization for the selected pre-trained CNN architectures.

Parameter	Resnet50	MobileNetV2	NASNetMobile	EfficientNetV2B0
Spatial dropout	0.2906	0.4667	0.3641	0.2153
Dense units	512	192	320	192
Dropout	0.6000	0.3163	0.3411	0.4999
Learning rate	1 × 10^−5^	6.457 × 10^−5^	9.0257 × 10^−5^	4.679 × 10^−5^

**Table 7:** Cohen’s Kappa index performance.

Value of Cohen’s Kappa	Level of Agreement
<0.00	Poor
0.00–0.20	Slight
0.21–0.40	Fair
0.41–0.60	Moderate
0.61–0.80	Strong
0.81–1.00	Almost perfect

**Table 8:** Training time of each CNN model selected from the best hyperparameter settings.

	Resnet50	MobileNetV2	NASNetMobile	EfficientNetV2B0
Time (Minutes)	48	41	54	44

**Table 9:** Overall confusion matrix-based evaluation metrics.

Metrics	Resnet50	MobileNetV2	NASNetMobile	EfficientNetV2B0
Accuracy	0.9658	0.9291	0.9584	0.9584
Precision	0.9730	0.9348	0.9674	0.9831
Recall	0.9524	0.9101	0.9418	0.9259
F1-Score	0.9626	0.9223	0.9544	0.9537

**Table 10:** Cohen’s Kappa values for inter-rater agreement across CNN models.

Model	Cohen’s Kappa Value
ResNet50	0.9310
MobileNetV2	0.8571
NASNetMobile	0.9162
EfficientNetV2B0	0.9160

## Data Availability

The datasets used in this study are publicly available from third-party sources. Two datasets were obtained from Roboflow Universe https://universe.roboflow.com/masters2022/corrosion-classification-v2 (accessed on 17 October 2025), https://universe.roboflow.com/zulfikar-setyo-priyambudi-hgsff/corrosion-classification-trvmp (accessed on 18 October 2025) and one dataset was obtained from Kaggle https://www.kaggle.com/datasets/ebrahim007/marine-corrosion-dataset (accessed on 18 October 2025).

## Abbreviations

The following abbreviations are used in this manuscript:

AI	Artificial Intelligence
API	Application Programming Interface
ANN	Artificial Neural Networks
CLAHE	Contrast-Limited Adaptive Histogram Equalization
CNN	Convolutional Neural Networks
GDP	Gross domestic product
Grad-CAM	Grad-CAM Gradient-weighted Class Activation Mapping
HSV	Hue Saturation Value
LMagNet	Lightweight Magnetic Convolutional Neural Network
ML	Machine Learning
MBConv	Mobile inverted Bottleneck Convolution
NDT	Non-Destructive Testing
PR-AUC	Precision–Recall Area Under the Curve
RAM	Random Access Memory
ROC-AUC	Receiver Operating Characteristic–Area Under the Curve
